# eEF1A Is an S-RNase Binding Factor in Self-Incompatible *Solanum chacoense*

**DOI:** 10.1371/journal.pone.0090206

**Published:** 2014-02-27

**Authors:** Jonathan Soulard, Nicolas Boivin, David Morse, Mario Cappadocia

**Affiliations:** Institut de Recherche en Biologie Végétale (IRBV), Département de Sciences Biologiques, Université de Montréal, Montréal, Québec, Canada; Chiba University, Japan

## Abstract

Self-incompatibility (SI) is a genetic mechanism that allows flowering plants to identify and block fertilization by self-pollen. In the Solanaceae, SI is controlled by a multiallelic *S*-locus encoding both S-RNases and F-box proteins as female and male determinants, respectively. S-RNase activity is essential for pollen rejection, and a minimum threshold value of S-RNases in the style is also required. Here we present biochemical evidence that eEF1A is a novel S-RNase-binding partner *in vitro*. We further show that the normal actin binding activity of eEF1A is enhanced by the presence of S-RNase. Lastly, we find that there is a co-localization of S-RNase and actin in the incompatible pollen tubes in structures reminiscent of the actin bundles formed by eEF1A. We propose that increased binding of eEF1A to actin in the presence of S-RNase could help explain the disruption of the actin cytoskeleton observed during SI reactions.

## Introduction

Angiosperms represent the largest and most diverse group of land plants. In most species flowers have male (anther) and female (pistil) sexual organs in close proximity, increasing the risk of self-pollination. To prevent selfing and limit the deleterious effects of inbreeding, angiosperms have adopted several strategies to promote out-crossing. Among these strategies, self-incompatibility (SI) is one of the most widespread, reported in more than 100 families and estimated to be present in almost 40% of species, and is considered an important factor of the evolutionary success of angiosperms [1]. SI is a genetically inherited device that allows a fertile flower to reject self- or closely related pollen by blocking the growth of pollen tubes on or inside the pistil, while genetically unrelated (non-self) pollen is accepted and can accomplish fertilization [2]. Solanaceae employ a gametophytic SI system (GSI), where the breeding behavior of the pollen is determined by its own haploid genotype. Both female and male specificity determinants of SI (style-S and pollen-S, respectively) are under the control of elements of a multigenic, multiallelic locus called the *S*-locus [2]. Variants of the *S*-locus are termed haplotypes, whereas variants of any of the individual genes in the *S*-locus are termed alleles [3].

The female determinant is a polymorphic glycoprotein with ribonuclease activity called the S-RNase [4] that is expressed in the style [5] and that penetrates inside the pollen tubes in a S-haplotype-independent manner [6]. The involvement of the S-RNase in SI has been firmly established in both loss- and gain-of-function transgenic plants [7], [8] and its catalytic activity is essential to its function [9]. The male determinant is expressed exclusively in the pollen and is determined by a group of S-locus-specific F-box proteins (termed variously SLF or SFB) acting collaboratively, each of which being responsible for the recognition and proteasome-mediated degradation of a subset of non-self S-RNases inside the pollen tube [10], [11]. Thus, in a compatible pollination, non-self pollen growth is permitted because the ensemble of SLF proteins present at its S-locus is able to degrade the S-RNases penetrating into the pollen tube [10]. Conversely, the rejection of self-pollen in an incompatible pollination is due to the fact that self S-RNases cannot be degraded by any of the SLF proteins of the pollen tube [10].

Full manifestation of the SI response also requires several other proteins not encoded by the S-locus (see [12]), some of which can interact directly with the *S*-RNases [13]. Among these, a stylar 11 kD protein, identified as a phytocyanin, was found to bind to the SC10-RNase from *Nicotiana alata* [14]. Another S-RNase-binding protein, SBP1, first found in *Petunia hybrida* pollen using a yeast-two-hybrid screen [15], was later shown to be present in many tissues and to interact with S-RNases in a non-S-haplotype-specific manner [15]–[17]. SBP1 contains a RING-HC domain in its C-terminal region suggesting it is involved in the ubiquitin-mediated degradation of S-RNases [15]. SBP1 also binds SLF proteins, and is thought to be part of a novel E3 ubiquitin-ligase complex able to target a specific S-RNase for degradation through its interaction with SLF, as found in *Petunia inflata* [16], [18]. Three stylar arabinogalactan proteins, NaTTS (*Nicotiana alata* transmitting tract specific), NaPELPIII (*Nicotiana alata* pistil extension-like protein III) and a 120 kDa glycoprotein have also been shown to interact with S-RNases immobilized on affinity resin [19], but no biochemical function for any is known. The 120 kDa glycoprotein in particular was found to be critical for SI [20] and to interact with SBP1 in a yeast-two-hybrid assay [21]. An additional protein reported to directly interact with S-RNases is NaTrxh, a secreted stylar protein belonging to the thioredoxin h subgroup II and identified by differential expression analysis in *Nicotiana*. NaTrxh, was found to bind and reduce S-RNases *in vitro* [22] although its involvement as a modifier gene in SI remains to be demonstrated. A *Petunia hybrida* ubiquitously expressed protein up-regulated in germinating pollen, PGPS/D3 [23], was also shown to bind S-RNases in a yeast-two-hybrid assay [24]. Finally, actin also interacts with S-RNases in a yeast-two-hybrid screen, although the interaction was not confirmed by pull-down experiments in native conditions [25]. This latter finding is intriguing since actin dynamics are an essential component of pollen tube growth [26], [27] and recent results suggest that S-RNases can act to disrupt the actin cytoskeleton in *Pyrus pirifolia* [28] and *Nicotiana alata* [29] pollen tubes.

The SI response also requires the presence of a minimum level of S-RNase in the style, an amount referred to as a threshold level. For example, transgenic plants must express more than this minimum level of S-RNase in order to show an SI phenotype, and several species show a phenomenon termed sporadic self-compatibility, or SSC (i.e. occasional fruit formation after crosses expected to be incompatible) (see [30] and references therein). In *Solanum chacoense*, SSC has been used to estimate the threshold for the S_12_-RNase in a number of different genotypes [30], [31], with compatibility correlated to below threshold levels. So far, no molecular mechanism has been proposed to account for the all-or-nothing SI response, which thus resembles an ultrasensitive response to S-RNase concentration.

In this work we report that eEF-1A, a component of the eukaryotic translational machinery, binds S-RNase *in vitro*. Furthermore, the normal actin binding of eEF1A is enhanced by the S-RNase. In addition, we observe the formation of actin bundles during incompatible crosses *in vivo*, and these bundles are often associated with S-RNase. We suggest that eEF1A may provide a functional connection between the S-RNase and the actin cytoskeleton. In addition, this finding may also help to explain the threshold phenomenon, as S-RNase sequestered in an eEF1A-actin complex may be unable to exercise its normal cytotoxic effects.

## Results

In Solanaceae-type gametophytic self-incompatibility (SI), glycosylated stylar ribonucleases, termed S-RNases, are taken up indiscriminately by growing pollen tubes [6]. Once inside the pollen tubes, interactions with proteins of the cytoplasm control S-RNase stability [16] and determine if they will be allowed to exert their cytotoxic action leading to the rejection of self-pollen tubes that share a common S-haplotype with the style [9]. To identify pollen proteins potentially involved in mediating the SI response, we first purified an S-RNase-enriched fraction from the styles of a *Solanum chacoense* genotype with an *S_11_S_12_* allelic composition. An immobilized lectin, Concanavalin-A (ConA) allows a range of glycoproteins to be purified from crude stylar extracts, of which the major component is the mono-glycosylated S_11_-RNase (Figure S1). The S_11_-RNase is more abundant than the S_12_-RNase [30] and is monoglycosylated, allowing it to be more easily eluted from the ConA column than the S_12_-RNase which has four glycosylation sites [32]. The partially purified glycoprotein fraction was then re-immobilized on ConA resin and used to select binding partners from a pollen extract. To ensure that only pollen proteins binding the immobilized glycoproteins were selected, the pollen extracts were themselves passed twice through ConA resin to remove any abundant proteins potentially binding the ConA alone. SDS-PAGE analysis of the retained proteins followed by LS-MS/MS sequencing identified a number of ribosomal proteins, and curiously, a translation factor called eEF1A (Table S1).

**Figure 1:**
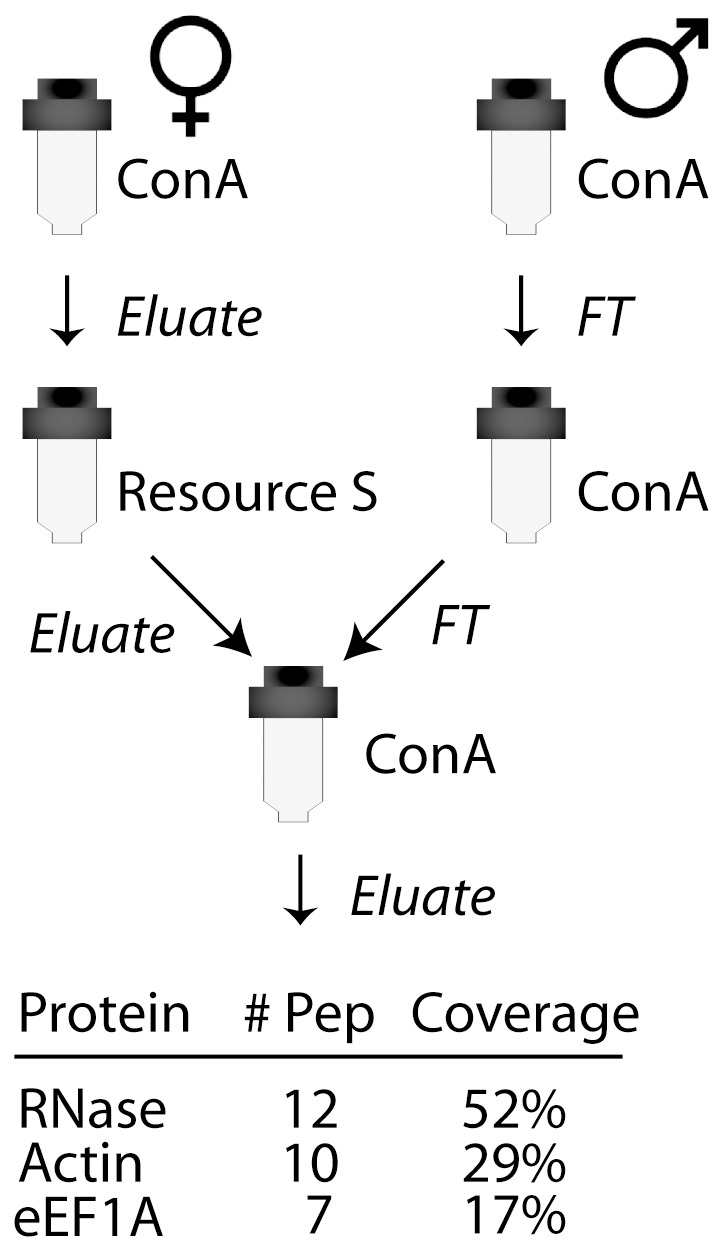
Protein purification schema and proteins identified in the final eluate. The stylar S-RNase was purified by ConA and Resource S (ion exchange) chromatography before being immobilized on ConA beads. Pollen proteins, depleted of proteins binding non-specifically to the ConA resin, were applied to the immobilized S-RNase, washed and eluted with ConA elution buffer. Only three proteins were detected in the specific eluate by LC-MS/MS.

The finding that a translation factor was an S-RNase binding partner was intriguing considering that the role of the S-RNase is to catalyze RNA degradation, thus blocking translation. To eliminate the possibility that a protein other than the S-RNase in the glycosylated protein fraction might be responsible for binding eEF1A, we prepared an even more highly purified S-RNase using ion exchange chromatography (IEX) to fractionate the conA-purified stylar extract prior to immobilization on a new ConA column (Figure 1). The entire specific eluate, analyzed directly by MS sequencing (Table S2), revealed that in addition to the S_11_-RNase only two proteins were present, eEF1A and actin. Thus, no other proteins appear to be involved in the interaction between these three components.

**Figure 2:**
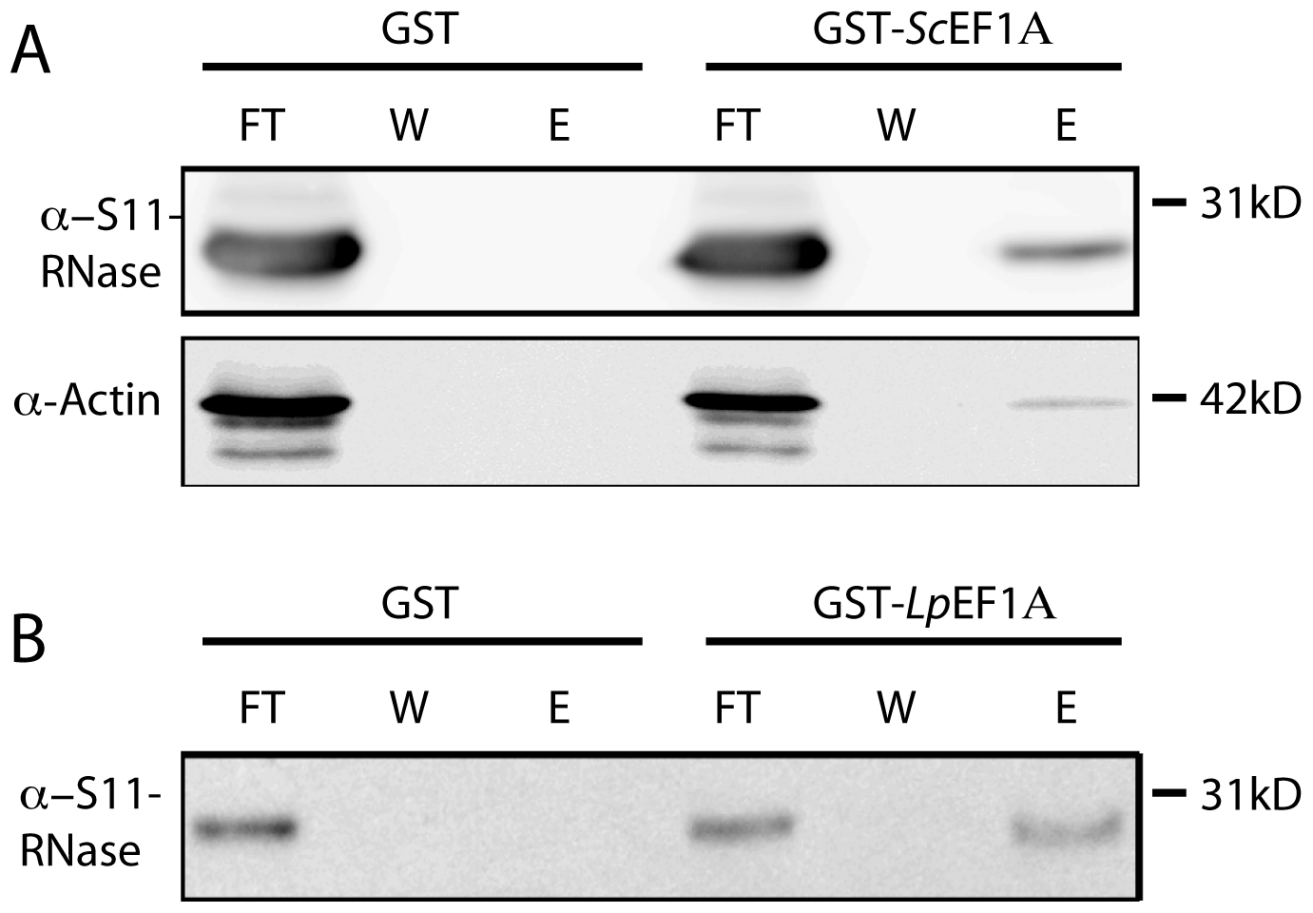
GST pull-down of S11-RNase by eEF1A. (A) Stylar total protein extracts containing an S11-RNase were used for chromatography on glutathione agarose beads in the presence of bacterially expressed GST (lanes 1–3) or with a bacterially expressed *S. chacoense* eEF1A-GST fusion protein (lanes 4–6). Samples for each experiment are 10% of the column flow-through (FT), the wash (W) and the specific eluate (E). Binding of the eEF1A to the S-RNase and to endogenous pollen actin was assessed by Western blot. (B) Binding of the S-RNase to a *Lingulodinium polyedrum* eEF1A.

To confirm the interaction between *S. chacoense* S_11_-RNase and eEF1A, we next immobilized eEF1A using a GST-tagged *S. chacoense* eEF1A expressed in bacteria. For this experiment, GST-eEF1A was purified using a glutathione agarose resin, then mixed with a total stylar protein extract and passed again through the affinity resin. Western blot analysis of the specific eluate showed that both S-RNase and actin were again retained on the immobilized eEF1A (Figure 2A). We also observed binding of the S-RNase to a heterologous eEF1A from *Lingulodinium polyedrum,* indicating S-RNase binding to eEF1A is not restricted to the *S. chacoense* protein (Figure 2B). No binding to GST alone was ever observed.

**Figure 3:**
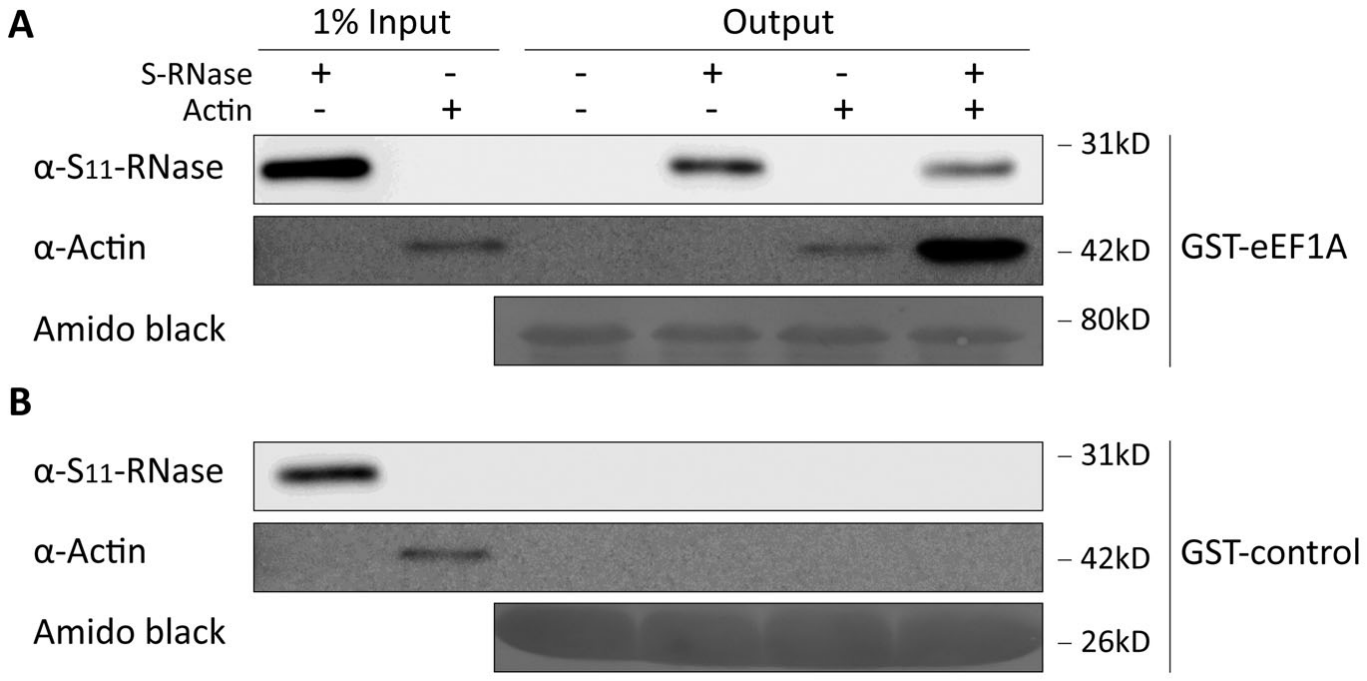
eEF1A binding to actin is increased by S_11_-RNase. (A) A GST-eEF1A fusion protein immobilized on GST resin was mixed with known amounts of purified S_11_-RNase and/or a commercial bovine actin. GST-eEF1A binds similar amounts of S_11_-RNase whether actin is present or not but the amount of actin bound by eEF1A increases markedly in the presence of S_11_-RNase. (B) A GST protein control binds neither S_11_-RNase nor actin.

Since actin accompanied S-RNase binding to eEF1A in this experiment, we were curious as to whether different pairs of proteins were able to bind one another. We first tested the ConA purified S-RNase immunologically for the presence of actin, reasoning that if binding between the two were possible, then actin should have co-purified with the S-RNase, but no actin could be detected (Figure S1). We next asked if our eEF1A could bind actin independently of the S-RNase, as widely documented in other systems [33] as well as bind the S-RNase independently of actin. We thus tested binding of a commercial actin and a purified S-RNase in different combinations to an immobilized GST-eEF1A. Immunological analysis of the specific eluates indicated that similar amounts of S-RNase were bound to eEF1A whether actin was present or not (Figure 3A, lanes 2, 4), suggesting that actin and S-RNase do not compete for the same binding site on eEF1A. However, while eEF1A alone bound moderate amounts of actin, the amount of actin bound was markedly increased by the presence of S-RNase (Figure 3B, lanes 3, 4). We interpret these results to indicate that the formation of a ternary complex between the S-RNase, EF1A and actin is likely to occur when all three proteins are present.

**Figure 4:**
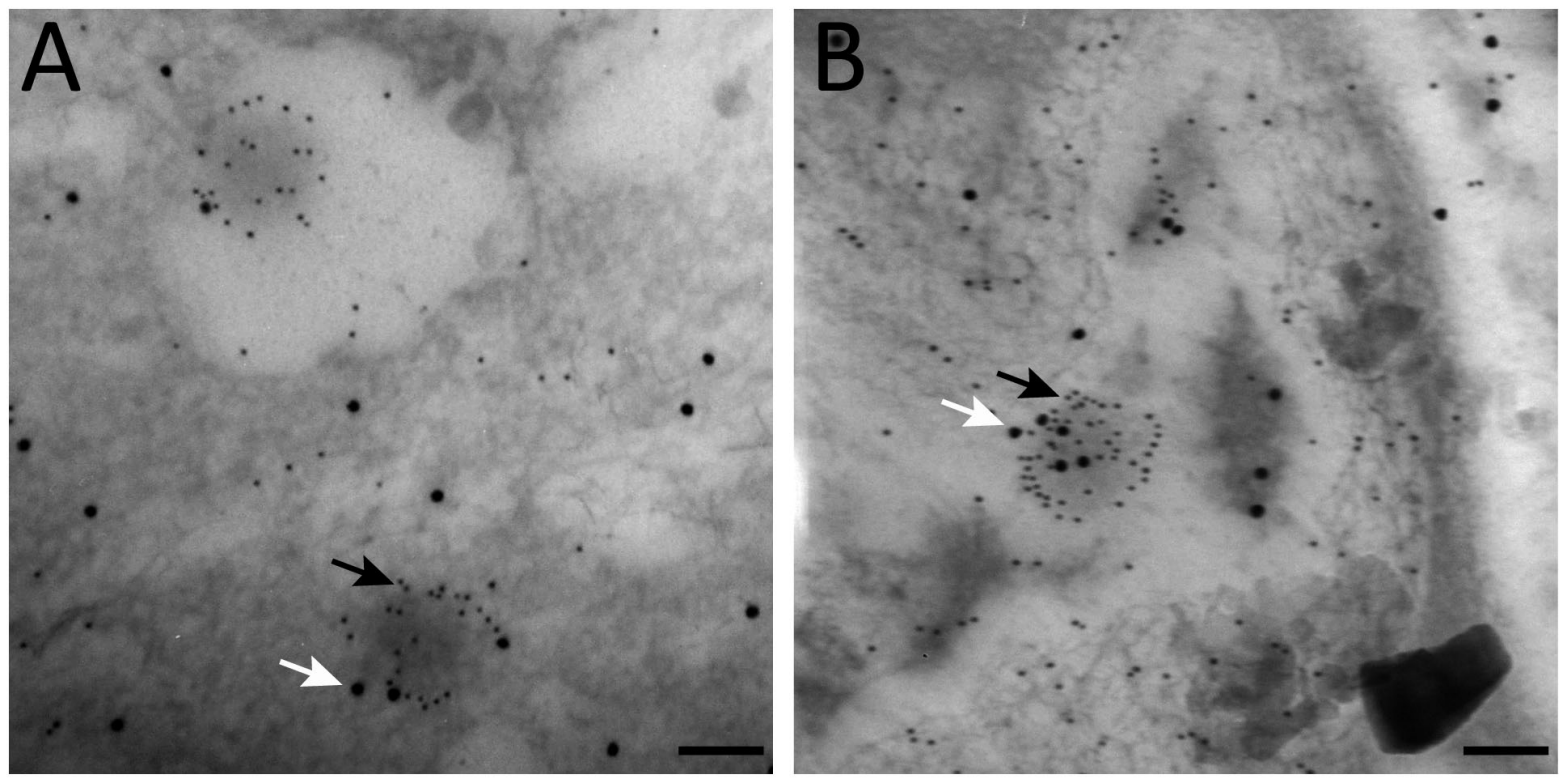
Regions of intense actin staining in incompatible pollen tubes also stain with S_11_-RNase. Incompatible pollen tubes 18(A) and 24 h (B) post-pollination stained simultaneously with anti-actin (5 nm colloidal gold, black arrows) and anti-S11-RNase (20 nm colloidal gold, white arrows). Scale bars are 0.1 µm.

Lastly, we were curious to see if any evidence could be found to support the interaction of S-RNase, eEF1A and actin *in vivo*. We therefore tested both our anti-S-RNase and the anti-actin on sections of styles taken either 18 or 24 hours post-pollination. If the ternary interaction observed *in vitro* was also occurring *in vivo*, we predicted that the S-RNase and actin should co-localize in pollen tubes. Interestingly, actin staining in incompatible pollen tubes was often found clustered in circular, electron-dense regions roughly 100 nm in diameter. These regions were always observed associated with S-RNase staining, generally to a degree higher than that found elsewhere in the cytoplasm (Figure 4).

## Discussion

eEF1A is one of several subunits that constitute the eukaryotic elongation factor eEF-1 [34]. In its GTP-bound form, eEF1A binds and delivers aminoacyl-tRNAs to the ribosome and thus regulates the elongation phase of protein synthesis [35]. eEF1A mRNA levels correlate with the rate of cell growth [36], although, as eEF1A is present in a **∼**20 fold molar excess over other elongation factors or the ribosome itself [37], it is unlikely to be the rate determining step [38]. This overabundance of eEF1A has led to suggestions that it may also be involved in other cellular processes, and indeed eEF1A has been shown to have “moonlighting” functions in nuclear export, proteolysis, apoptosis and signal transduction [34]. One of its most highly documented features, however, is its interaction with the actin cytoskeleton and its bundling activity on actin filaments [39]–[41], a role that appears to be mutually exclusive with its function in translation [42]. First reported in *Dictyostelium* [43], this activity can be regulated by pH [44] and Ca^2+^/Calmodulin [45], [46] and has been suggested to be evolutionary conserved [39]. eEF1A is considered to be essential for the regulation of the actin cytoskeleton [47], which reciprocally can influence protein synthesis [48]. Interestingly, both protein synthesis and the cytoskeletal structure are affected during the S-RNase-based gametophytic self-incompatibility response [28], [29], [49].

During the growth of pollen tubes in the style, S-RNases are imported from the extracellular matrix of the style into the pollen tube cytoplasm in a *S*-haplotype-independent manner [6]. The cytotoxic effect of the S-RNases, and the subsequent incompatibility reaction, occurs when pollen tubes share the same haplotype as the S-RNase [2]. Interestingly, it has recently been shown that S-RNases might also trigger disruption of the actin cytoskeleton in self-incompatible pollen tubes [28], [29]. By contrast, in compatible pollen tubes, S-RNases are targeted for degradation [50], which abrogates their cytotoxic action. Curiously, a minimum threshold quantity of S-RNase in the style is required to trigger the self-incompatibility reaction [30], [32], [51], but to date, no mechanism has yet been proposed to account for this. The threshold phenomenon is conceptually akin to a sigmoidal or ultrasensitive response for which a variety of mechanisms, including co-operativity, positive feedback loops and molecular titration [52] have been proposed. It is an intriguing possibility that eEF1A may potentially act to titrate the amount of free S-RNase following import into the pollen tube cytoplasm. If true, this binding could give rise to an ultrasensitive response because the amount of unbound S-RNase is likely to increase most markedly when the cytoplasmic supply of eEF1A is exhausted. The eEF1A concentration would thus define the inflection point in the ultrasensitive response to increasing S-RNase concentrations. Interestingly, the binding and immobilization of the S-RNase in a complex with eEF1A and actin might also result in sequestration of eEF1A, which in turn might prevent its subsequent participation in protein synthesis.

During normal pollen tube elongation, the actin cytoskeleton is organized as an array of long filaments extending from the base to a distal region of the pollen tube just behind the growing tip, where the extensive vesicular activity required for membrane extension occurs [53]. The integrity of the cytoskeletal organization and in particular its dynamic nature is critical to the polarized growth of the pollen tube [26]. During the incompatible reaction, the presence of self S-RNases results in a gradual disorganization of the filamentous actin cytoskeleton and in a reduction in the pollen tube growth rate [29]. This drastic effect on actin reorganization is not seen in compatible pollen tubes, possibly due to degradation of the S-RNase. It is thus tempting to speculate that recruitment of the S-RNase-eEF1A complex to the actin cytoskeleton may provide a potential functional link between the stylar incompatibility component and the extensive reorganization of the actin cytoskeleton that occurs during the incompatible reaction [28], [29]. Indeed, the staining of incompatible pollen tubes with anti-actin is suggestive of bundles whose size is consistent with the bundles formed *in vitro* by the eEF1A from *Dictyostelium* [43], [44]. In our case, such bundles also contain S-RNase, although S-RNase labeling unassociated with the areas of intense actin staining is also observed in the cytoplasm.

Our results demonstrate a previously unsuspected binding between eEF1A and S-RNases. This binding is unlikely to be simply due to electrostatic attraction since eEF1A and the S_11_-RNase are both basic proteins (pI 9.5 and 8.4 respectively). Furthermore, this binding does not require actin although the amount of actin binding to eEF1A in our pull-down experiments is stimulated markedly by the presence of S-RNase (Figure 3). We note that our purified S-RNase preparations do not contain immunologically detectable levels of actin (Figure S1), implying that appreciable binding of S-RNase to actin requires eEF1A. Our results thus suggest models where either all three proteins bind together or where eEF1A acts as an intermediate between the S-RNase and actin. It must be noted that several reports in the literature suggest a possible binding between actin and mammalian or fungal ribonucleases such as bovine seminal RNase [54], angiogenin [55] or ACTIBIND [56]. The nature of this binding is unclear, however, as the native structure and activity of at least the fungal enzyme is not required [56]. Actin has also recently been suggested as a candidate protein interacting with the *Prunus avium* S_6_-RNase in a yeast two-hybrid screening [25], but subsequent pull-down assays only validated such interaction at concentrations of reducing agents high enough to potentially induce protein aggregation [57].

In summary, we have identified eEF1A as a potential S-RNase-binding protein in *S. chacoense* pollen extracts. We propose that binding between the two may provide an explanation for the S-RNase threshold effect observed in the self-incompatibility reaction, below which pollen rejection does not occur [30], [32], [51]. Moreover, this finding opens up a new and tantalizing glimpse of how S-RNases may influence the actin cytoskeleton and translation in pollen tubes. This opens a fascinating vista of potential rejection mechanisms of the pollen tubes by the S-RNase, considering both the actin cytoskeleton and the translation apparatus, which are increasingly shown to be interdependent, are disrupted during the self-incompatibility reaction.

## Methods

### Plant Material

The self-incompatible diploid genotypes (2n = 2x = 24) of *Solanum chacoense* used in this study include the previously described L25 and V22 lines carrying the self-incompatibility alleles *S_11_S_12_* and *S_11_S_13_* respectively [31], as well as the *S_12_S_12_* line 2548 [50].

### Protein Extraction and Purification

Stylar proteins were extracted from 0.5 g of L25 styles ground in liquid nitrogen using ConA extraction buffer (100 mM Tris HCl pH 7.5, 50 mM NaCl, 5 mM EDTA, 0.1% 2-mercaptoethanol). After clarification by centrifugation, the extraction buffer was replaced with ConA binding buffer (100 mM Tris HCl pH 7.5, 500 mM NaCl, 1 mM MgCl_2_, 1 mM MnCl_2_, 1 mM CaCl_2_, 0.02% 2-mercaptoethanol) by chromatography on PD-10 columns (GE Healthcare Biosciences, PA) and the extract incubated with 0.5 mL of Concanavalin A resin (GE Healthcare Biosciences, PA) at room temperature for 1 h. After washing the resin with ConA binding buffer, the bound proteins were washed with low-salt ConA binding buffer (100 mM Tris HCl pH 7.5, 100 mM KCl, 1 mM MgCl_2_, 1 mM MnCl_2_, 1 mM CaCl_2_, 0.02% 2-mercaptoethanol). The bound proteins were then either used directly for the pollen protein binding assay on the resin or were eluted using ConA elution buffer (100 mM Tris HCl pH 7.5, 500 mM NaCl, 5 mM EDTA, 0.02% 2-mercaptoethanol, 1 M α-D-methyl-glucoside) for further purification. To further purify the S-RNase, the buffer in the ConA eluate was replaced with IEX binding buffer (50 mM MES pH 5.5) by PD-10 chromatography and the extract applied to a Resource S cation-exchange column using an AKTA FPLC system (GE Healthcare Biosciences, PA). Proteins were eluted with a linear gradient from 0 to 1 M NaCl in IEX binding buffer, and all fractions containing the S_11_-RNase, as verified by immunoblotting, were pooled. The IEX elution buffer was then replaced with ConA binding buffer by PD-10 chromatography and the purified S_11_-RNase extract immobilized on 0.5 mL of ConA resin. The resin was then washed with low-salt ConA binding buffer before use for the pollen protein-binding assay.

Pollen proteins were extracted from 0.5 g of mature L25 pollen in ConA extraction buffer using a French press. After clarification by centrifugation, the extraction buffer was replaced with low-salt ConA binding buffer and the sample incubated twice with 0.5 mL of Concanavalin A resin to deplete Concanavalin A-binding proteins from the pollen extract. Unbound proteins were then incubated with the ConA-bound stylar proteins or ConA-bound purified S_11_-RNase at room temperature for 1 h. The resin containing bound protein was subsequently washed with low-salt ConA binding buffer and ConA binding buffer. Proteins remaining bound to the resin were then eluted with ConA elution buffer. Protein fractions were then electrophoresed on SDS-PAGE and stained with Coomassie Blue. Candidate protein bands binding a partially purified S_11_-RNase extract were excised and sequenced separately by LC-MS/MS at the Institut de Recherche en Immunologie et Cancérologie (IRIC, Université de Montréal), whereas the entire eluate containing proteins binding the Resource S-purified S_11_-RNase was sequenced directly.

### Cloning and Recombinant Protein Expression

A full length *S. chacoense* eEF1A coding sequence (GenBank Accession Number KF573426) was cloned and sequenced using sequence information derived from Illumina sequencing of pollen RNA from lines V22 and 2548 using BamHI-ScEF1a-F (5′ GGGGGGATCCGGTAAGGAAAAGATTCACAT 3′) as a forward primer and ScEF1a-BamHI-R (5′ GGGGGGATCCTCACTTTCCCTTCTTCTGGG 3′) as a reverse primer. The sequence was subcloned into the BamHI site of the pGEX-4T-2 protein expression vector (GE Healthcare Biosciences, PA) in frame with the N-terminal GST tag. The derived amino acid sequence of this pollen tube eEF1A contains all the amino acids determined by MS sequencing of the ConA column eluate. An eEF1A coding sequence from the dinoflagellate *Lingulodinium polyedrum* was also amplified using primer sequences derived from a sequence database generated by Illumina RNA-Seq (GenBank Accession number JO706501), forward primer BamHI-EF1a-F (5′ GGGGGGGGATCCTCTGATCAAAAAGAACATGTCTCTA 3′) and reverse primer Ef1a-NotI-R (5′ GGGGGGGCGGCCGCTTATTCAATCTTTGTAATTTTACCA 3′) and using *L. polyedrum* cDNA as template. This sequence was similarly subcloned in pGEX-4T-2 to create a N-terminal GST fusion protein.

The expression vectors were used to transform BL21(DE3) competent *E. coli* (New England Biolabs, MA) according to the manufacturer’s instructions. For protein expression, 100 mL of each culture at an OD of 0.5 were induced with 0.1 mM IPTG and incubated for 3 hours at 28°C. Cultures were lysed in extraction buffer (PBS pH 8.0, 0.1% Triton X-100, 5 mM DTT and 2 mM PMSF) using a French press. The GST-tagged recombinant proteins were purified by incubating clarified crude bacterial extracts for 2 h with 200 µL GST resin (Bio Basic Inc, Ontario). After washing the resin with the same buffer, bound proteins were eluted using elution buffer (50 mM Tris HCl pH 8.0, 50 mM NaCl, 0.1% Triton X-100, 1 mM DTT and 10 mM reduced glutathione). The buffer in the eluted proteins was replaced with interaction buffer (50 mM Tris HCl pH 8.0, 50 mM sodium chloride, 0.1% Triton X-100, 1 mM DTT) by chromatography on PD-10 columns (GE Healthcare Biosciences, PA). The protein concentration of the purified extracts was determined using a Bio-Rad Protein Assay kit (Bio-Rad Laboratories, CA).

### GST Pull-down Experiments

For pull-downs using only ConA purified S-RNase (as in Figure 2), stylar proteins were extracted from 0.25 g of V22 styles ground in liquid nitrogen using GST extraction buffer (PBS pH 8, 0.1% Triton X-100, 5 mM DTT and 2 mM PMSF) and the extraction buffer replaced with GST interaction buffer using PD-10 columns. Typically, 1 mg stylar protein was incubated with 100 µL of GST resin preloaded with either 250 µg GST or 250 µg of the GST-tagged eEF1A for 2 h at room temperature. The resin then washed four times with GST interaction buffer before elution of the bound proteins with interaction buffer containing 10 mM reduced glutathione. All the protein fractions were precipitated with 4 volumes of acetone and resuspended in 100 µl of SDS-PAGE loading buffer. Sample volumes corresponding to 5% of the flow through or wash and 40% of the eluate were electrophoresed on SDS-PAGE and transferred on a Hybond C-extra nitrocellulose membrane (GE Healthcare Biosciences, PA) using a Trans-Blot SD Semi-Dry Electrophoretic Transfer Cell (Bio-Rad Laboratories, CA) according to the manufacturer’s instructions. Protein transfer was visualized by staining the membranes with Ponceau S and recorded using an ImageQuant LAS 4000 imaging system (GE Healthcare Biosciences, PA). For the pull-downs using Resource S purified S-RNase (as in Figure 3), the GST resin was loaded with GST or GST-tagged eEF1A as above. This resin was then incubated for 1 h at room temperature with either 10 µg of purified S_11_-RNase, 10 µg of a commercial bovine actin preparation (Sigma-Aldrich Corporation, MO) or both together. The resin was then washed and eluted as above.

### Immunoblotting Analyses

Antibodies for western blots [58] included a rabbit anti-S_11_-RNase antibody [58], a commercial plant-specific mouse monoclonal anti-actin (Sigma-Aldrich Corporation, MO) and a commercial animal specific rabbit anti-actin (Sigma-Aldrich Corporation, MO), used as described previously [59] or according the manufacturer’s instructions. Primary antibodies were detected using commercial horseradish-peroxidase (HRP)-conjugated secondary antibodies (Sigma-Aldrich Corporation, MO) and the Immobilon Western Chemiluminescent HRP substrate kit (EMD Millipore Corporation, MA) according the manufacturer’s instructions and recorded using an ImageQuant LAS 4000 imaging system system.

### Immunoelectron Microscopy

Immunolocalization experiments with a 1/50 dilution of a rabbit anti-S_11_-RNase were performed as described using a 20 nm colloidal gold-labeled goat anti-rabbit secondary antibody [6]. Co-localization with actin was assessed by simultaneous labeling with a 1/50 dilution of a commercial mouse anti-actin (Sigma-Aldrich Corporation, MO) visualized using a 5 nm colloidal gold-labeled goat anti-mouse secondary antibody.

## Supporting Information

Figure S1 **S11-RNase-enriched preparations by ConA chromatography do not contain actin.** A partially purified S11-RNase fraction eluted from a ConA column contains the S11-RNase as the principal component. Stylar actin does not co-purify with the S11-RNase as shown by the lack of anti-actin antibody staining in the ConA eluate.
(JPG)

Table S1 **Proteins interacting with a crude S-RNase preparation.**
(DOCX)

Table S2 **List of peptides sequenced from proteins interacting with an immobilized highly purified S-RNase.**
(DOCX)
