# Supplementary figures and images for: eEF1A Is an S-RNase Binding Factor in Self-Incompatible Solanum chacoense
Source: PLoS One. 2014 Feb 27;9(2):e90206. doi: 10.1371/journal.pone.0090206 (PMC3937366; doi:10.1371/journal.pone.0090206)

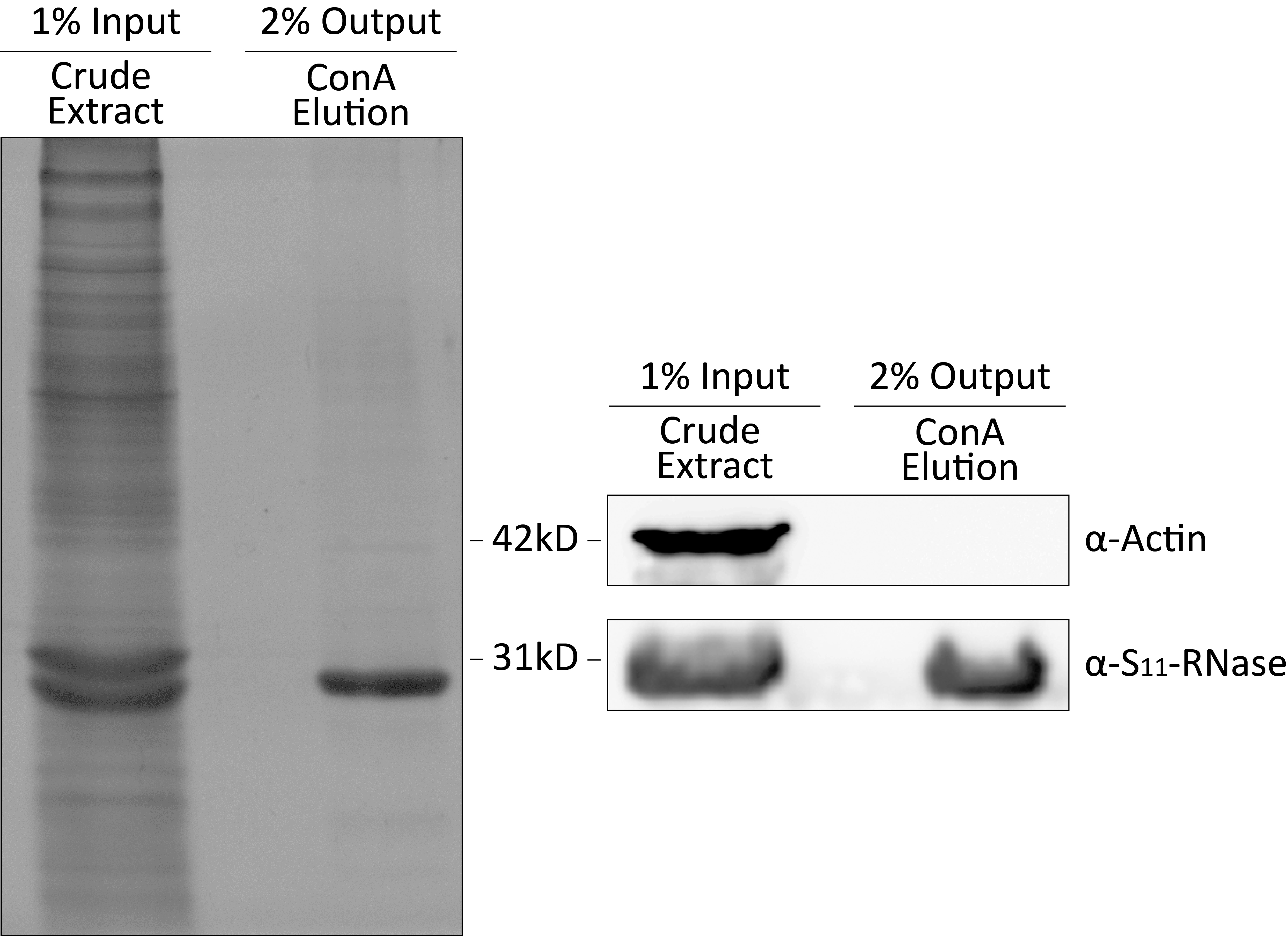

Supplement: Figure S1 — S11-RNase-enriched preparations by ConA chromatography do not contain actin. A partially purified S11-RNase fraction eluted from a ConA column contains the S11-RNase as the principal component. Stylar actin does not co-purify with the S11-RNase as shown by the lack of anti-actin antibody staining in the ConA eluate. (JPG) [file pone.0090206.s001.jpg]
