# Supplementary material for: eEF1A Is an S-RNase Binding Factor in Self-Incompatible Solanum chacoense
Source: PLoS One. 2014 Feb 27;9(2):e90206. doi: 10.1371/journal.pone.0090206 (PMC3937366; doi:10.1371/journal.pone.0090206)
Supplement: Table S1 — Proteins interacting with a crude S-RNase preparation. (DOCX) [file pone.0090206.s002.docx]

Table S1

Proteins interacting with a crude S-RNase preparation.

MW_obs_ Best match Coverage MW_exp_

(kDa) (*S. tuberosum*) (%) (kDa)

50 eEF1A 26 50

49 60S ribosomal protein L4-1 45 45

48 60S ribosomal protein L4-1 51 45

40 60S ribosomal protein L4-1 42 45

36 60S ribosomal protein L5 29 35

33 60S ribosomal protein L7A-like 44 29

32 60S ribosomal protein L7A-like 44 29

30 60S ribosomal protein L7 56 28

28 60S ribosomal protein L7 45 28
