# Supplementary material for: eEF1A Is an S-RNase Binding Factor in Self-Incompatible Solanum chacoense
Source: PLoS One. 2014 Feb 27;9(2):e90206. doi: 10.1371/journal.pone.0090206 (PMC3937366; doi:10.1371/journal.pone.0090206)
Supplement: Table S2 — List of peptides sequenced from proteins interacting with an immobilized highly purified S-RNase. (DOCX) [file pone.0090206.s003.docx]

**Table S2.** List of peptides sequenced from proteins interacting with an immobilized highly purified S-RNase.

| **S-RNase** |  |  |
| --- | --- | --- |
| Position | Predicted Peptides | Recovered Peptides |
| 3 | MFK |  |
| 26 | SLLTSTLFIVLFSLSSTYGDFDK |  |
| 45 | LQLVLTWPPSFCHANNCQR | LQLVLTWPPSFCHANNCQR |
| 49 | IVPK |  |
| 60 | NFTIHGLWPDK |  |
| 67 | EGPQLLK | EGPQLLK |
| 72 | YCKPK |  |
| 81 | LTYNYFSDK | LTYNYFSDK |
| 88 | MLNDLDK | MLNDLDKHWIQLK |
| 94 | HWIQLK | HWIQLK |
| 101 | IDQASAR | IDQASAR |
| 102 | K |  |
| 108 | DQPAWK | KDQPAWK |
| 113 | YQYLK | YQYLK |
| 120 | HGSCCQK |  |
| 133 | IYNQNTYFSLALR | IYNQNTYFSLALR |
| 135 | LK |  |
| 137 | DR |  |
| 142 | FDLLR |  |
| 148 | TLQIHR | TLQIHR |
| 165 | IVPGSSYTFEEIFDAVK | IVPGSSYTFEEIFDAVK |
| 175 | TVTQMDPDIK | TVTQMDPDIK |
| 199 | CTEGAPNLYEIGICFTPNGDSLVR |  |
| 201 | CR |  |
| 208 | QSETCDK |  |
| 211 | TGK |  |
| 216 | IFFRP |  |
|  |  |  |
| **Actin** |  |  |
| Position | Predicted Peptides | Recovered Peptides |
| 20 | MADGEDIQPLVCDNGTGMVK |  |
| 30 | AGFAGDDAPR |  |
| 41 | AVFPSIVGRPR | AVFPSIVGRPR |
| 52 | HSGVMVGMGQK | HTGVMVGMGQK |
| 63 | DAYVGDEAQSK | DAYVGDEAQSK |
| 64 | R |  |
| 70 | GILTLK |  |
| 86 | YPIEHGIVSNWDDMEK |  |
| 97 | IWHHTFYNELR |  |
| 115 | VAPEEHPVLLTEAPLNPK | VAPEEHPVLLTEAPLNPK |
| 118 | ANR |  |
| 120 | EK |  |
| 149 | MTQIMFETFNTPAMYVAIQAVLSLYASGR |  |
| 179 | TTGIVLDSGDGVSHTVPIYEGYALPHAILR |  |
| 185 | LDLAGR |  |
| 193 | DLTDSLMK | DLTEHLAK |
| 198 | ILTER |  |
| 208 | GYSFTTSAER | GYSFTTSAEK |
| 212 | EIVR |  |
| 215 | DVK |  |
| 217 | EK |  |
| 233 | LAYIALDYEQELETSK |  |
| 240 | TSSSVEK |  |
| 256 | SYELPDGQVITIGAER | NFELPDGQVITIGAER |
| 258 | FR |  |
| 286 | CPEVLFQPSMIGMEAAGIHETTYNSIMK |  |
| 292 | CDVDIR | CDVDIR |
| 293 | K |  |
| 314 | DLYGNIVLSGGTTMFPGIADR |  |
| 317 | MSK |  |
| 328 | EITALAPSSMK | EITALAPSSMK |
| 330 | IK |  |
| 337 | VVAPPER | VVAPPER; VVAPPERK |
| 338 | K |  |
| 361 | YSVWIGGSILASLSTFQQMWIAK |  |
| 374 | AEYDESGPSIVHR |  |
| 375 | K |  |
| 377 | CF |  |
|  |  |  |
| **EF1A** |  |  |
| Position | Predicted Peptides | Recovered Peptides |
| 3 | MGK |  |
| 5 | EK |  |
| 20 | IHISIVVIGHVDSGK |  |
| 30 | STTTGHLIYK |  |
| 36 | LGGIDK |  |
| 37 | R |  |
| 41 | VIER |  |
| 44 | FEK |  |
| 51 | EAAEMNK |  |
| 52 | R |  |
| 55 | SFK |  |
| 62 | YAWVLDK |  |
| 64 | LK |  |
| 67 | AER |  |
| 69 | ER |  |
| 79 | GITIDIALWK |  |
| 84 | FETTK |  |
| 96 | YYCTVIDAPGHR |  |
| 100 | DFIK |  |
| 129 | NMITGTSQADCAVLIIDSTTGGFEAGISK |  |
| 134 | DGQTR |  |
| 146 | EHALLAFTLGVK |  |
| 154 | QMICCCNK |  |
| 161 | MDATTPK |  |
| 164 | YSK |  |
| 166 | AR |  |
| 172 | YDEIVK |  |
| 179 | EVSSYLK | EVSSYLK |
| 180 | K |  |
| 187 | VGYNPDK | VGYNPDKIPFVPISGFEGDNMIER |
| 204 | IPFVPISGFEGDNMIER |  |
| 212 | STNLDWYK | STNLDWYK |
| 227 | GPTLLDALDQINEPK |  |
| 235 | RPTDKPLR |  |
| 243 | LPLQDVYK | LPLQDVYK |
| 254 | IGGIGTVPVGR | IGGIGTVPVGR |
| 278 | VETGVIKPGMVVTFGPTGLTTEVK |  |
| 301 | SVEMHHEALLEALPGDNVGFNVK |  |
| 306 | NVAVK | NVAVKDLK |
| 309 | DLK |  |
| 310 | R |  |
| 318 | GFVASNSK |  |
| 323 | DDPAK |  |
| 359 | GAASFTAQVIIMNHPGQIGNGYAPVLDCHTSHIAVK |  |
| 366 | FAEILTK |  |
| 369 | IDR |  |
| 370 | R |  |
| 373 | SGK |  |
| 377 | ELEK |  |
| 380 | EPK |  |
| 383 | FLK |  |
| 391 | NGDAGMVK |  |
| 411 | MIPTKPMVVETFAEYPPLGR |  |
| 415 | FAVR |  |
| 418 | DMR |  |
| 427 | QTVAVGVVK | QTVAVGVVK |
| 431 | NVDK |  |
| 432 | K |  |
| 438 | DPTGAK |  |
| 441 | VTK |  |
| 445 | AAQK |  |
| 446 | K |  |
| 448 | GK |  |
